# Perforation of Meckel’s diverticulum in two neonates with single umbilical artery

**DOI:** 10.1136/wjps-2024-000770

**Published:** 2024-05-08

**Authors:** Lifeng Zhang, Shannan Wu, Xuefeng Miao, Yonglin Li, Xiaojian Yuan, Zhigang Gao

**Affiliations:** 1 General Pediatric Surgery, Children's Hospital, Zhejiang University School of Medicine, Hangzhou, China; 2 Department of Pediatric Surgery, Yiwu Branch of Children's Hospital, Zhejiang University School of Medicine, Yiwu, China

**Keywords:** Pediatrics, Neonatology

Meckel’s diverticulum (MD) is the most common congenital anomaly of the gastrointestinal (GI) tract. The main clinical manifestation of MD in childhood is GI bleeding. However, symptomatic manifestation in the neonatal period is rare.^1^ Single umbilical artery (SUA) was first reported by Hyrtl in 1870.^2^ In recent years, SUA has been reported to be a marker for fetal congenital malformations, chromosomal abnormalities, premature birth, and low birth weight, which can lead to severe adverse outcomes. We hereby report two cases of MD perforation in neonates with SUA in the Yiwu Branch of Children’s Hospital, Zhejiang University School of Medicine.

Case 1 was a female neonate who was born at more than 31 weeks of gestation. Prenatal ultrasound (US) showed an ‘LV’ shape in the transverse section and a single spiral structure in the longitudinal section of the free segment of the fetal umbilical cord, which indicated SUA. Chromosome examination of the fetus was normal, and no obvious structural abnormalities were detected on prenatal US. On March 8, 2021, the infant was delivered by cesarean section for threatened premature labor and growth restriction, and was diagnosed with SUA during delivery. The neonate’s birth weight was 1010 g, and the Apgar score was 9’−10’/1–5 points. The neonate developed dyspnea 26 minutes after delivery and was admitted to the neonatal intensive care unit for neonatal respiratory distress syndrome. The next day, the newborn developed abdominal distension and progressively worsened. The abdominal radiograph obtained with the patient in the upright position showed a large amount of free gas in the abdominal cavity (figure 1), which indicated GI perforation. Case 2 was a male neonate who was born at more than 34 weeks of gestation. Prenatal US also indicated SUA. The neonate was delivered by cesarean section for premature rupture of membranes for 36 hours at another hospital on April 10, 2022, and was diagnosed with SUA during delivery. The neonate’s birth weight was 2090 g, and the Apgar score was 9’−10’/1–5 points. After breast feeding on the day of birth, the infant developed vomiting and bloating, and radiograph obtained with the patient in the upright position also indicated GI perforation. The infant was then transferred to our hospital.

**Figure 1:**
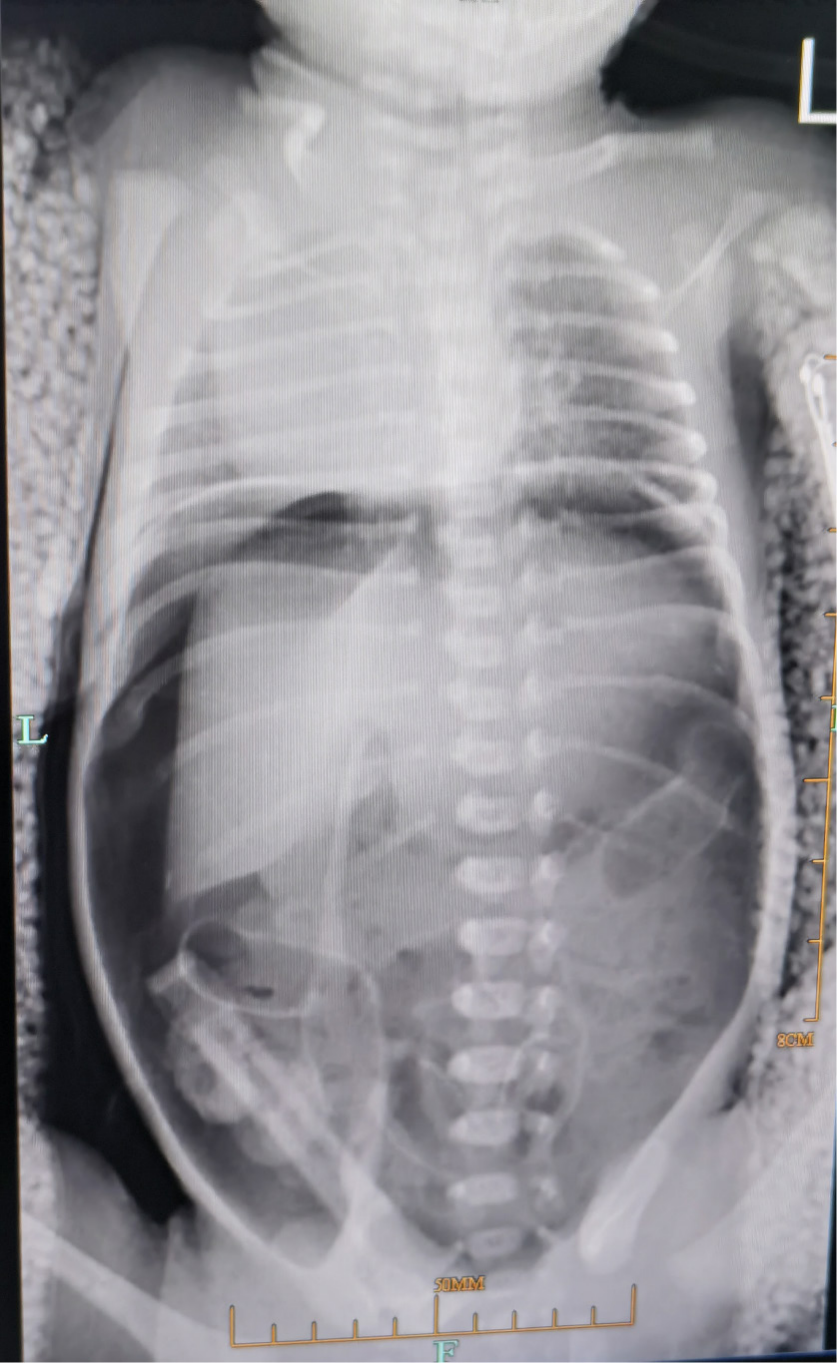
Abdominal radiograph of the female patient in the upright position.

Both neonates were fasted and treated with GI decompression, oxygen therapy, incubator, fluid replacement, anti-infective therapy, and preoperative preparations. Emergency laparotomy was performed in both neonates, which confirmed the presence of MD perforation. The diverticulum in both cases was located on the opposite side of the mesentery of the terminal ileum, approximately 20 cm and 25 cm from the ileocecal valve, respectively. The base width of the diverticula in both cases was approximately 0.6 cm and 0.8 cm, and the length was approximately 0.8 cm and 1.0 cm, respectively. The perforation in both cases was located at the top of the diverticulum, where the wall was blackened, collapsed, and necrotic, with a diameter of approximately 0.4 cm. In both cases, there was no obvious swelling at the base of the diverticulum. The surrounding intestinal tube was mildly adhered. There was no meconium-stained in the abdominal cavity and no extensive intestinal adhesions in either case (figure 2).

**Figure 2:**
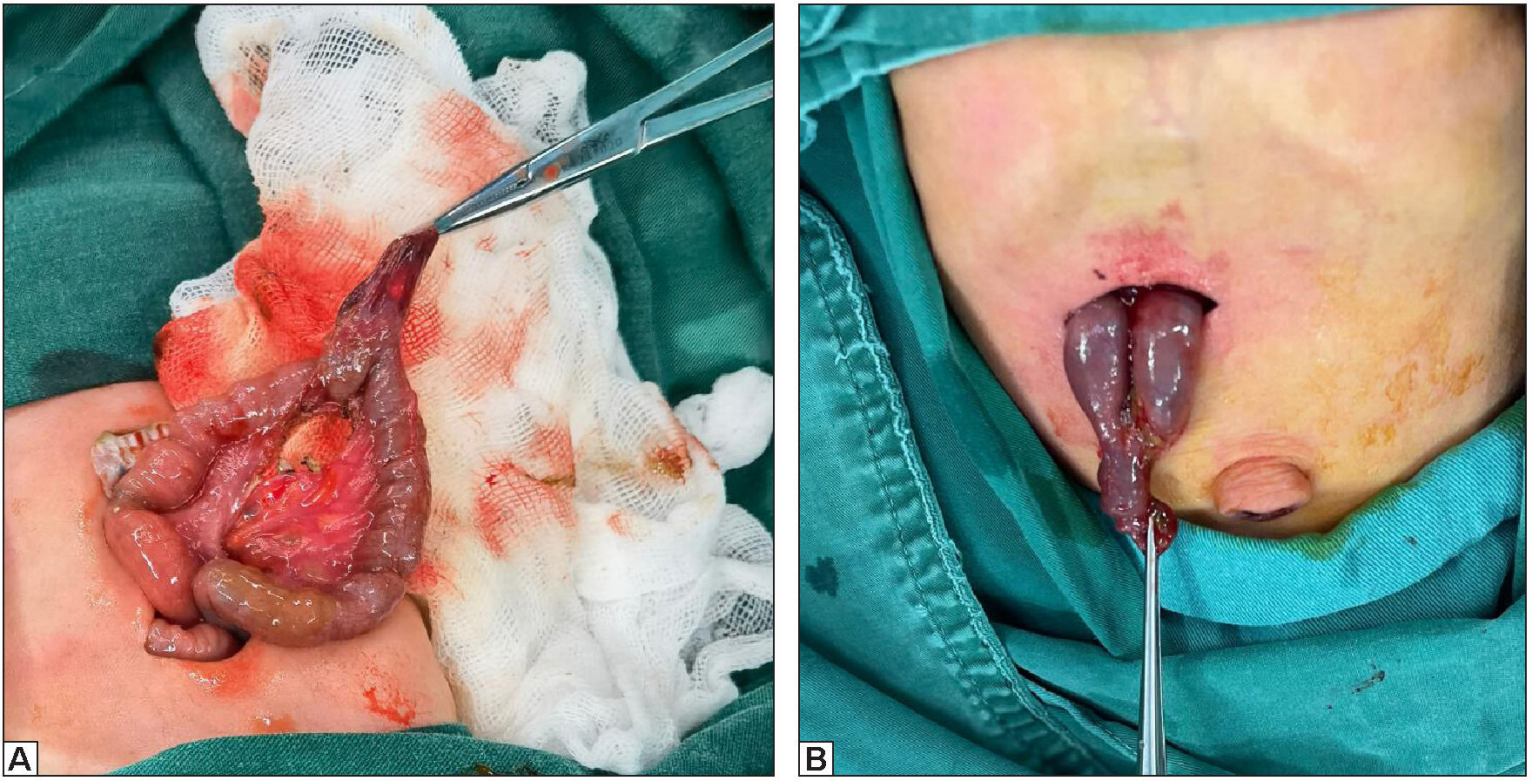
Intraoperative images of cases 1 (A) and 2 (B) show perforation of small intestinal diverticulum.

Both neonates underwent resection of the diverticulum and the attached intestinal segment, followed by intestinal anastomosis. Pathological examination of the resected diverticula revealed that both cases of diverticula had intestinal wall structures, lined with intestinal mucosa, localized bleeding or necrosis. In addition, many inflammatory cells were infiltrated in the diverticula and partial muscular layer defect at the top of the diverticula, but no ectopic tissue was observed. Both patients recovered after surgery and were discharged without complications. The patients were followed up for 1.5 years and 0.5 years. Both patients had normal growth and development, no GI abnormalities occurred, and the prognosis was satisfactory.

Symptomatic MD is very rare in neonates, with only a few reported cases of MD perforation in neonates found in literature searches conducted over the past 5 years.^1 3–8^ In this study, we report two cases of neonatal MD, both of which presented with GI perforation. SUA is one of the most common prenatal diagnoses in cases of fetal abnormality. Fetuses with SUA are at risk of increased chromosomal abnormalities and congenital malformations.^9 10^ The most widely accepted underlying explanations for the cause of SUA include primary agenesis of one umbilical artery, subsequent thrombotic atrophy of one umbilical artery or persistence of the original single allantoic artery of the body stalk.^2^ SUAs can be either of the primary agenesis type or the later thrombotic atrophy type. Different types of SUAs have the same effect on embryonic development. Therefore, the causes and types of formation of SUA are not yet well understood, which can be further studied in the future.

This is the first report of MD perforation in neonates with SUA. The incidence of structural abnormalities in fetuses with SUA is higher, suggesting that there may be a relationship between SUA and MD formation; however, the precise mechanism involved remains unclear. Possible explanations may include abnormal degeneration of the yolk sac during early embryonic cord formation, or impaired maternal–fetal exchange due to SUA, which affects the growth and development of the fetal digestive system. Perforation of MD is common in children and is mostly caused by inflammation. The perforation of MD in newborn is very rare, and we found from the pathology that the cause of this perforation was muscular defect, which is obviously different from the perforation mechanism of MD in children. However, we do not have clinical case data on whether the perforation of MD in neonates with SUA is different from that in neonates with non-SUA. Further studies can be conducted if more cases are found. Physicians should consider the possibility of MD perforation in cases of SUA in neonates with early digestive tract perforation. Some cases of neonatal MD perforation may be associated with local muscular layer defects in the diverticulum. Therefore, the perforation of MD in neonates may be related to the partial muscular layer defect in the diverticula.

## Data Availability

Data are available upon reasonable request.
